# Supplementary material for: A Mott insulator continuously connected to iron pnictide superconductors
Source: Nat Commun. 2016 Dec 19;7:13879. doi: 10.1038/ncomms13879 (PMC5187431; doi:10.1038/ncomms13879)
Supplement: Supplementary Information — Supplementary Figures 1-12, Supplementary Tables 1-3, Supplementary Notes 1-5 and Supplementary References. [file ncomms13879-s1.pdf]

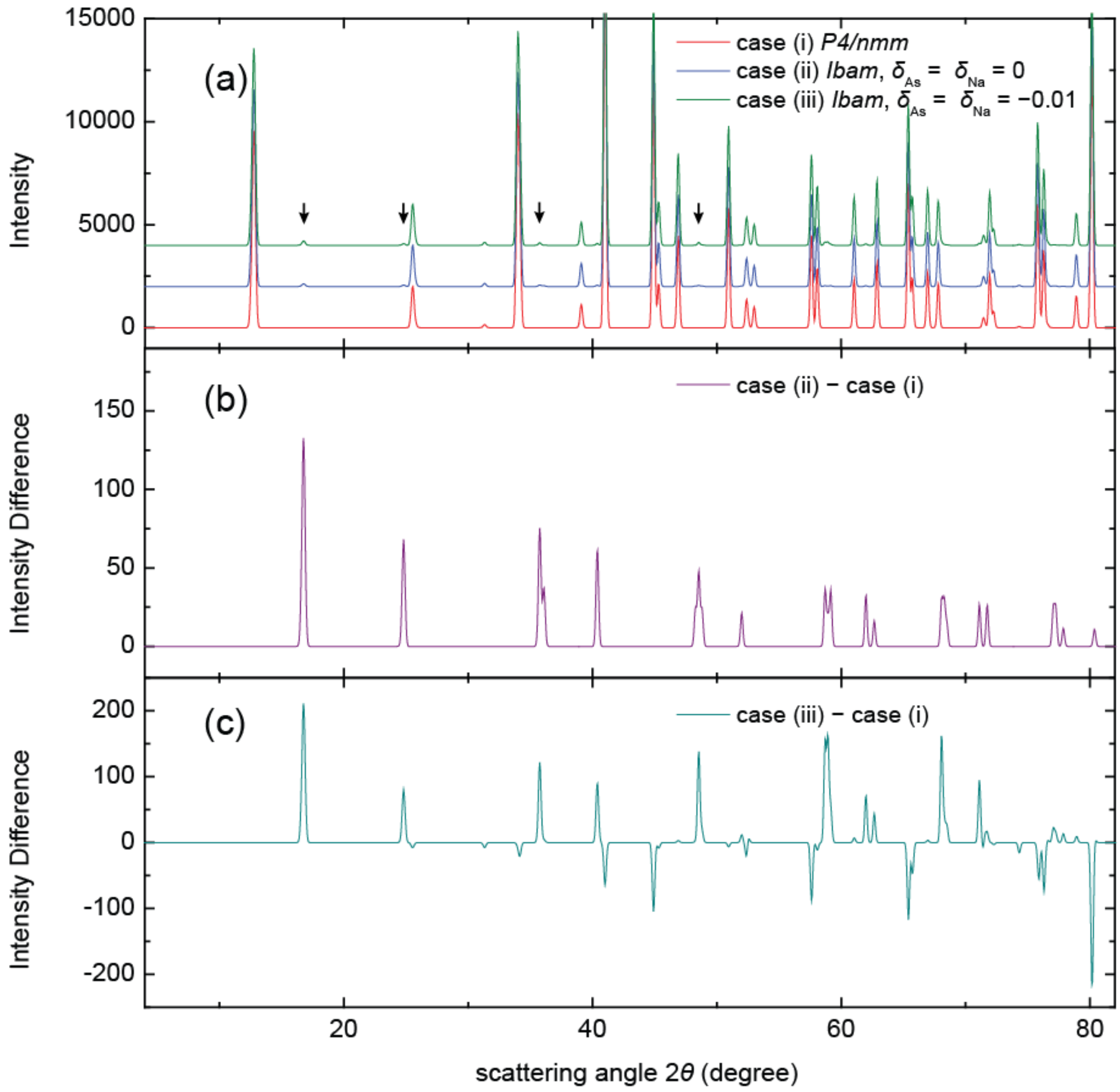

Supplementary Figure 1:Calculated neutron powder diffraction profiles for $NaFe_{0.5}Cu_{0.5}As$.(a) Case (i) where Fe and Cu are completely disordered and $\delta_{As} = \delta_{Na} = 0$, corresponding to the *P4/nmm*space group when $a = c$ (*Ibam* notation). Case (ii) has Fe-Cu stripe order and $\delta_{As} = \delta_{Na} = 0$, case (iii) further has non-zero δ_{As} and δ_{Na}. The black arrows mark some super-lattice peak positions. (b) Difference between case (ii) and case (i), when Fe and Cu order into stripes, only super-lattice peaks are formed without affecting nuclear Bragg peaks already present in case (i). (c) Difference between case (iii) and case (i), having non-zero δ_{As} and δ_{Na}only slightly affects the intensities of nuclear peaks already present in case (i). Note the intensity range is much smaller in (b) and (c) compared to (a).

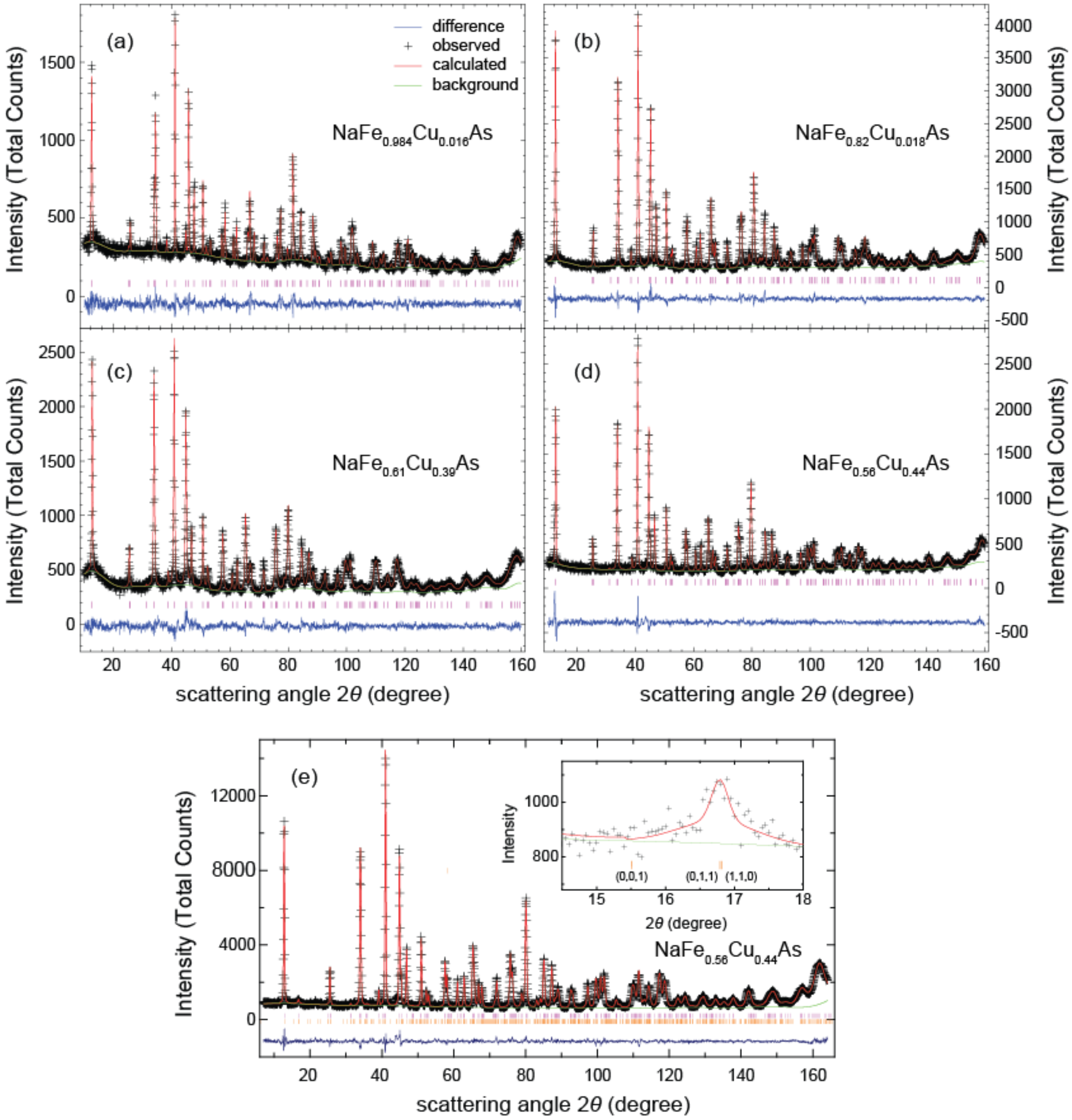

Supplementary Figure 2: Neutron powder diffraction data for $NaFe_{1-x}Cu_xAs$ measured at 300K and 4K. (a) Observed (black crosses) and calculated (red lines) neutron powder diffraction intensities for $NaFe_{0.984}Cu_{0.016}As$ at 300 K using space group *P4/nmm*. Short magenta vertical lines represent nuclear Bragg peak positions, the blue trace is the difference between measured and calculated intensities. (b), (c) and (d) show similar results for $NaFe_{1-x}Cu_xAs$ with x = 0.18, 0.39 and 0.44 respectively. Despite the observation of super-lattice peaks in samples with x = 0.39 and 0.44 at 300K, such peaks are not visible in neutron powder diffraction data and all data sets at 300K are fit with the same tetragonal structure as undopedNaFeAs in the paramagnetic state with space group *P4/nmm*. (e) $NaFe_{0.56}Cu_{0.44}As$ at 4K fit with *Ibam* structure described in Supplementary Table 1 and the magnetic structure in Fig. 1(d), magenta vertical lines correspond to nuclear Bragg peak positions and orange vertical lines are magnetic Bragg peak positions. The inset zooms in on a super-lattice/magnetic peak.

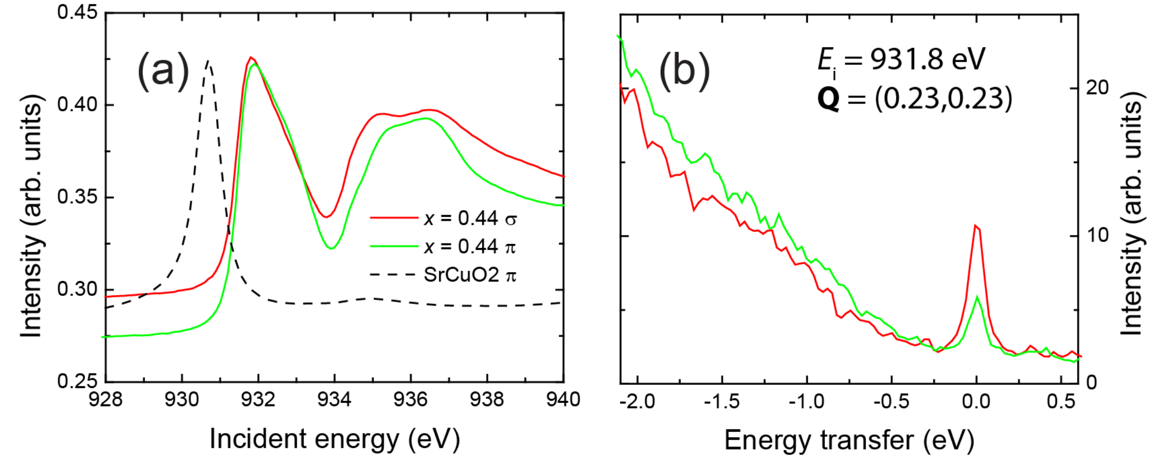

Supplementary Figure 3:XAS and RIXSmeasurements on $NaFe_{0.56}Cu_{0.44}As$. (a) X-ray absorption spectroscopy (XAS) measurements on $NaFe_{0.56}Cu_{0.44}As$ (solid lines) and $SrCuO_2$ (dashed line) obtained at 10 K. (b) Typical resonant inelastic X-ray scattering (RIXS) measurement on the same compound with incident energy E_i = 931.8 eV and in-plane momentum transfer $q_{//}$ = 0.498 $Å^{-1}$ obtained at 10 K. Light green lines are measurements with π polarization and red lines are corresponding measurements with σ polarization.

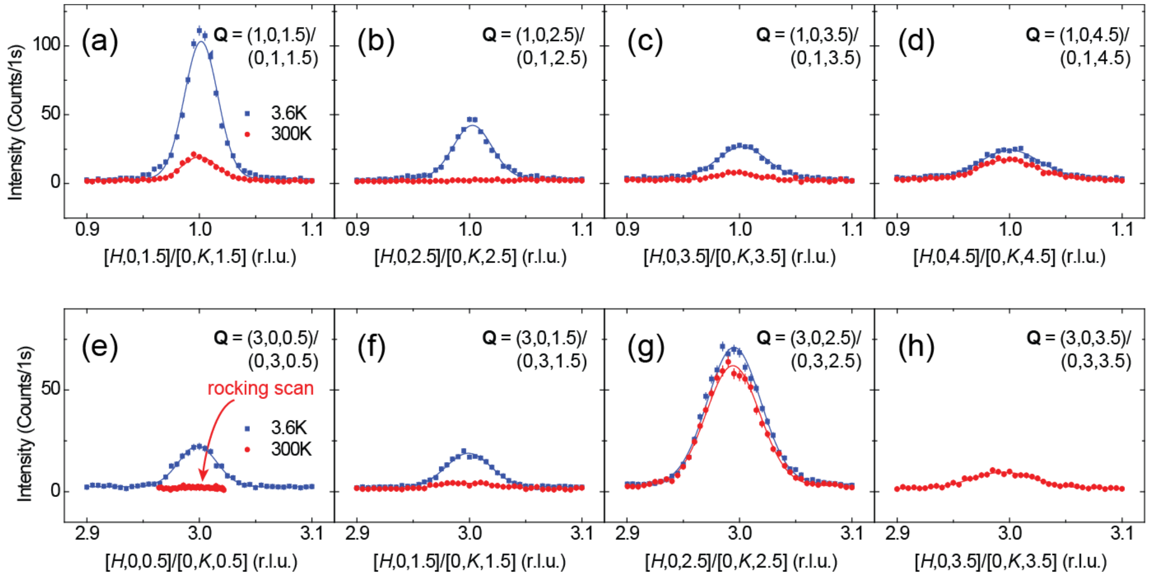

Supplementary Figure 4:Scans along [*H*, 0, *L*]/[0,*K*,*L*]at wave-vectors equivalent to (1,0,0.5)/(0,1,0.5) for $NaFe_{0.56}Cu_{0.44}As$ at 3.6 K and 300 K.These scans were measured with unpolarized neutrons. Scans at 3.6 K are shown as blue squares and scans measured at 300 K are shown as red circles. Scans centered at **Q** = (1, 0, *L*)/(0,1,*L*) with *L* = 1.5, 2.5, 3.5 and 4.5 are respectively plotted in (a), (b), (c) and (d). Similarly scans centered at **Q**= (3, 0, *L*)/(0,3,*L*) with *L* = 0.5, 1.5, 2.5 and 3.5 are shown in (e), (f), (g) and (h), respectively. Scan along [*H*, 0, 0.5]/[0,*K*,0.5] at 300 K for **Q** = (3, 0, 0.5)/(0,3,0.5) was not measured, instead a rocking scan is plotted to show that there is no observable intensity at this wave-vector and temperature. Scan along [*H*, 0, 3.5]/[0,*K*,3.5] at 3.6 K centered at **Q** = (3, 0, 3.5)/(0,3,3.5) was not measured. All vertical error bars represent statistical error (1 s. d.).

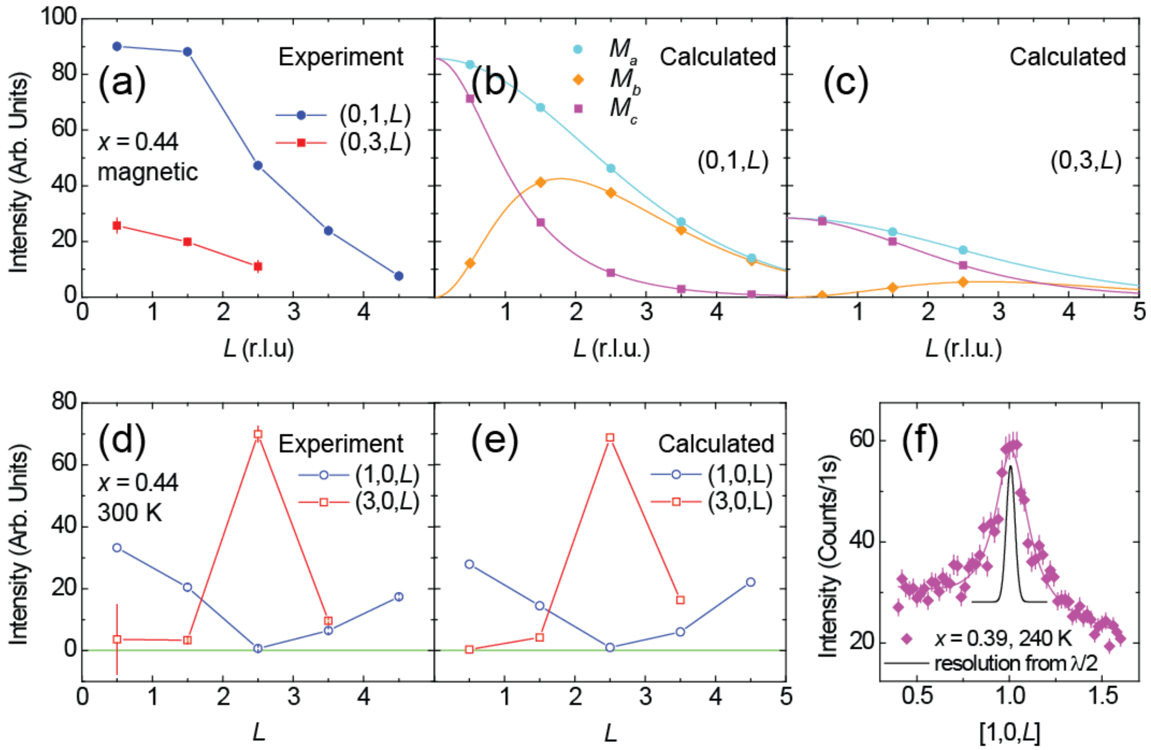

Supplementary Figure 5: Wave vector dependence of super-lattice peaks and magnetic peaks. (a) The magnetic intensities for equivalent wave-vectors (0, 1, *L*) (solid blue circles) and (0, 3, *L*) (solid red squares) are summarized for $NaFe_{0.56}Cu_{0.44}As$. The intensities were obtained from rocking scans at 3.6 K, correcting for contributions from super-lattice peaks estimated from [*H*, 0, *L*]/[0,*K*,*L*] scans presented in Supplementary Figure 4. (b) Assuming Fe^{2+}magnetic form factor, the expected intensities for (0, 1, *L*) wave-vectors are shown depending on the orientation of the magnetic moment. The magnetic moment along *a* axis (orange diamonds), *b* axis (cyan circles) and *c* axis (magenta squares) are compared. (c) Expected magnetic intensities are similarly shown for (0,3,*L*). (d) Super-lattice peak intensities for equivalent wave-vectors (1, 0, *L*) (empty red squares) and (3, 0, *L*) (empty blue circles) obtained from scans at 300 K in Figure S4, (e) shows the corresponding calculated intensity for $NaFe_{0.56}Cu_{0.44}As$ using the structure in Supplementary Table 1. (f) Scan along the [1, 0, *L*] direction for $NaFe_{0.61}Cu_{0.39}As$ at 240 K (magenta diamonds), the instrument resolution (black line) is obtained by performing the same scan after removing the filters. Vertical error bars in (a), (d) and (e) are from least-square fits (1 s. d.). Vertical error bars in (f) represent statistical error (1 s. d.).

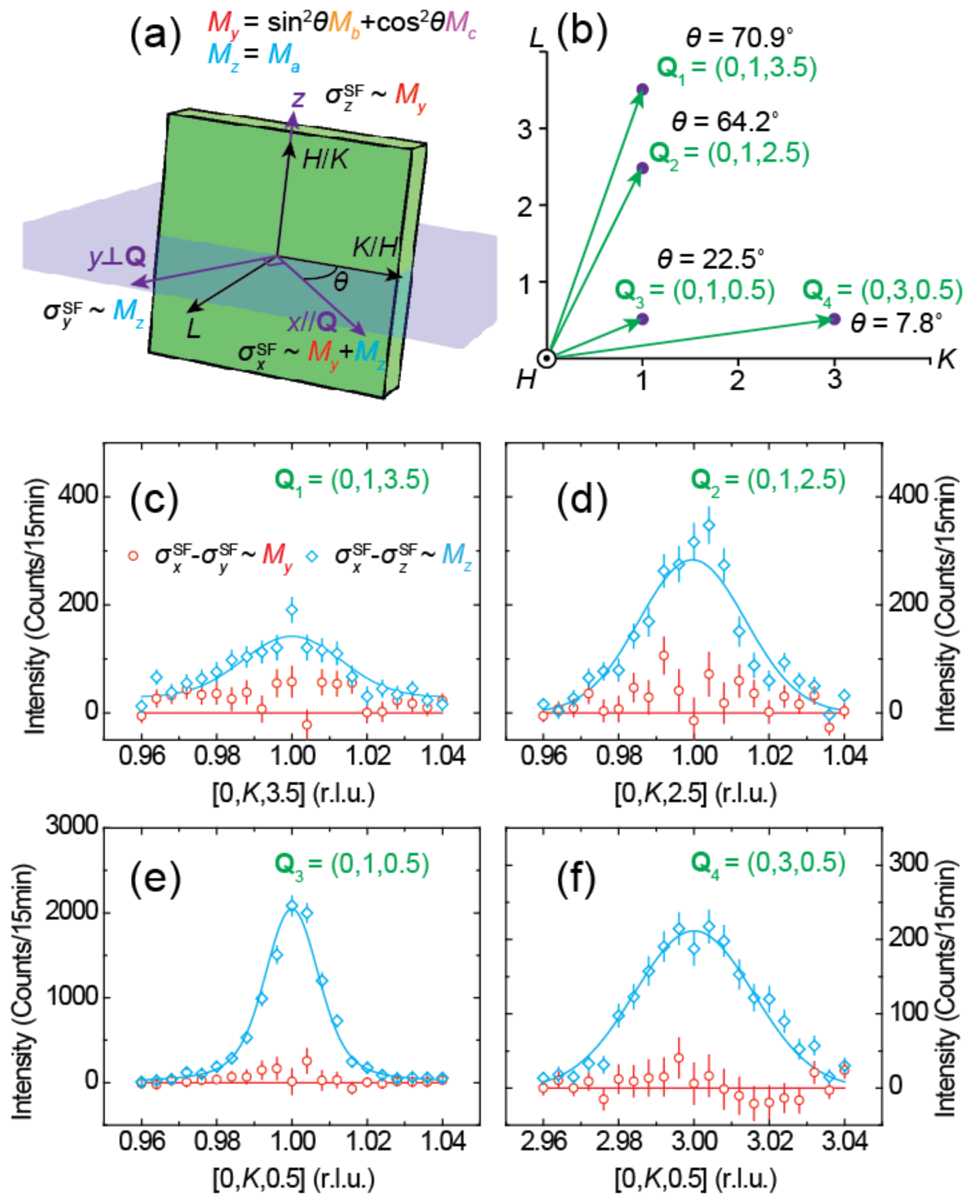

Supplementary Figure 6: Determining the spin direction using polarized neutron scattering. (a) Definition of x, y and z directions with respect to the crystallographic axes. (b) Wave vectors probed in [0,K,L] scattering plane and the correspondingθ. M_y (open red circles) and M_z (open cyan diamonds) for$\mathbf{Q}_1$ = (0,1,3.5), $\mathbf{Q}_2$ = (0,1,2.5), $\mathbf{Q}_3$ = (0,1,0.5) and $\mathbf{Q}_4$ = (0,3,0.5) are shown in (c), (d), (e) and (f), respectively. All vertical error bars represent statistical error (1 s. d.).

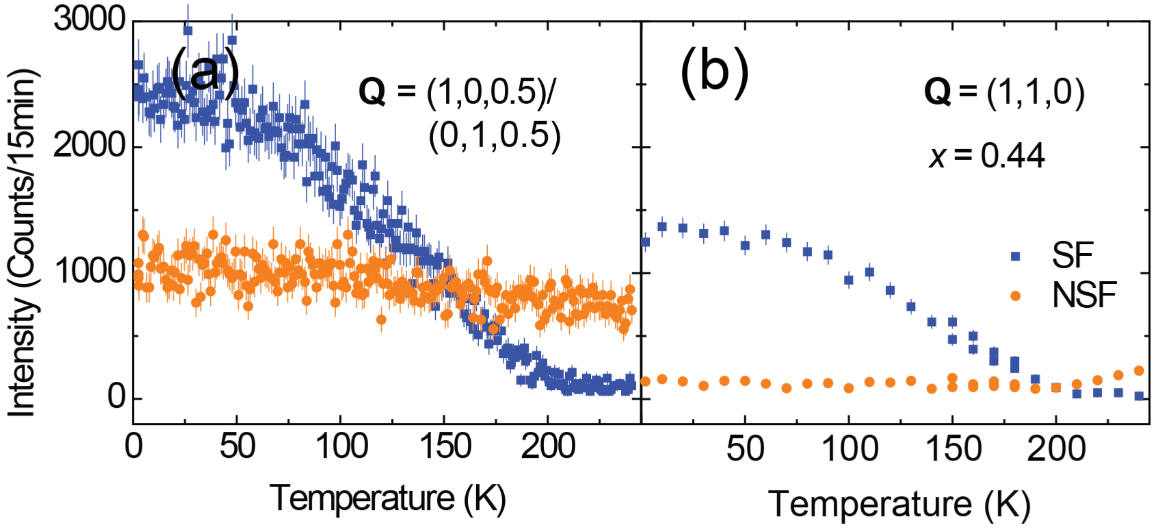

Supplementary Figure 7: Temperature dependence of magnetic and super-lattice peaks in $NaFe_{1-x}Cu_xAs$. (a) Temperature dependence of SF (blue squares) and NSF (orange circles) channels for $NaFe_{0.56}Cu_{0.44}As$ at **Q** = (1,0,0.5)/(0,1,0.5). Magnetic signal coming from **Q** = (0,1,0.5) is seen in the SF channel whereas super-lattice signal coming from ***Q*** = (1,0,0.5) are seen in the NSF signal. (b) Similar temperature dependence of SF and NSF channels at **Q** = (1,1,0). All vertical error bars represent statistical error (1 s. d.).

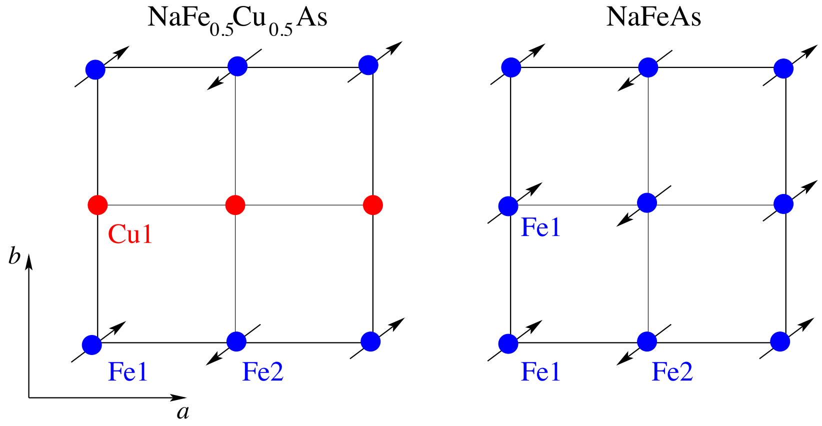

Supplementary Figure 8 |Schematic depiction of the in-plane AF ordering in $NaFe_{0.5}Cu_{0.5}As$ (left panel) used by the electronic structure calculations.Rows of Cu atoms (red) turn out to carry no magnetic moment, whereas spins of Fe atoms (blue) take on the arrangement similar to the parent compound NaFeAs (right panel) with the in-plane ordering wave-vector **Q** = (1,0) in the orthorhombic notation. The rows of Cu atoms alternate in the *c*-direction, according to the experimentally determined *Ibam*spacegroup. Note that the spin polarization axis is depicted symbolically and is not representative of the actual spin orientation relative to the crystalline axes.

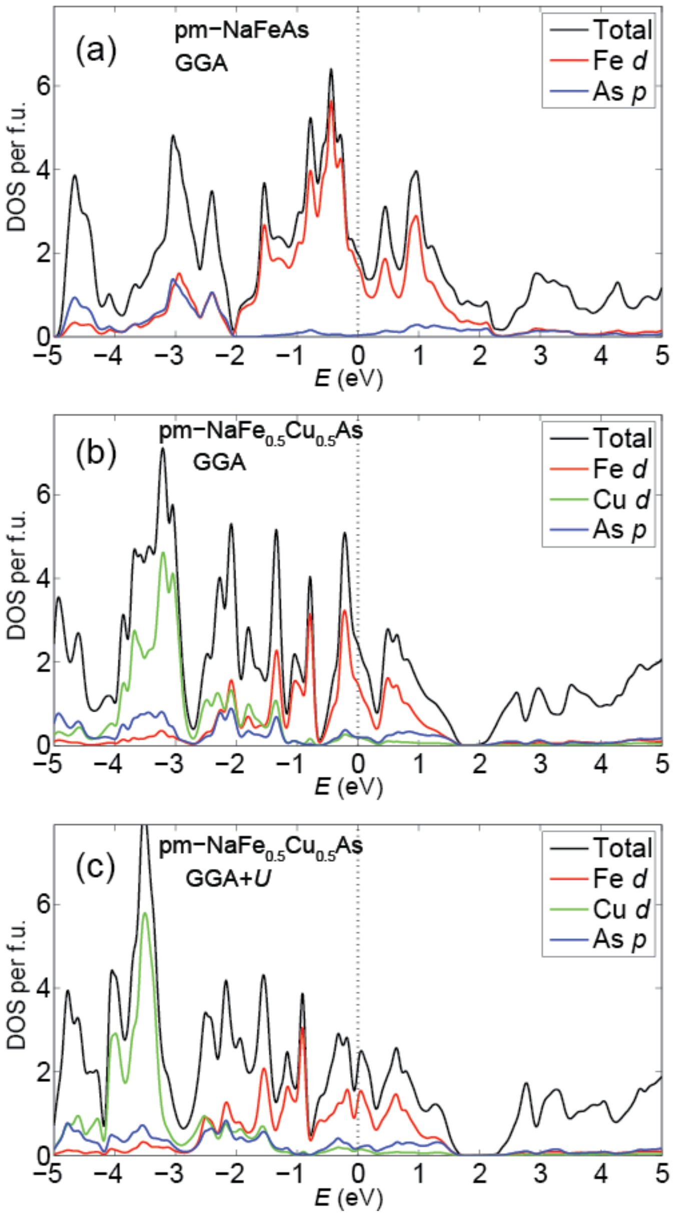

Supplementary Figure 9:Electronic density of states (DOS) calculated with the DFT-GGA method. The paramagnetic phase of (a) parent material NaFeAs, (b) $NaFe_{0.5}Cu_{0.5}As$. Partial density of states due to *d*-electrons of Fe and Cu are shown, together with the As*p*-electron contribution. The zero of energy is chosen to be at the Fermi level, indicated with a vertical dashed line. (c) Attempt to capture the effect of the Coulomb repulsion in paramagnetic $NaFe_{0.5}Cu_{0.5}As$ results in a metallic ground state within the DFT+*U* method [see Supplementary Note 4 for details].

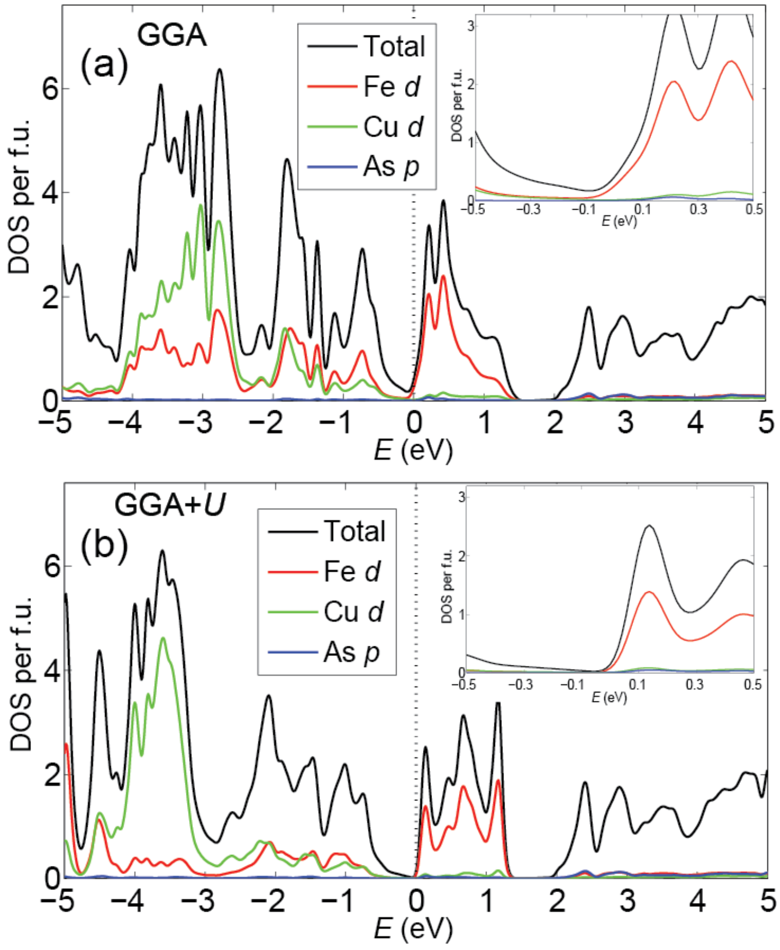

Supplementary Figure 10:Electronic density of states in the antiferromagnetically ordered phase of $NaFe_{0.5}Cu_{0.5}As$ calculated with (a) DFT-GGA method, and (b) DFT+U method. Partial contribution to the density of states due to *d*-electrons of Fe and Cu and *p*-electrons of As are shown. The insets show the zoomed-in details of the DOS near the Fermi level (denoted by a dashed line).

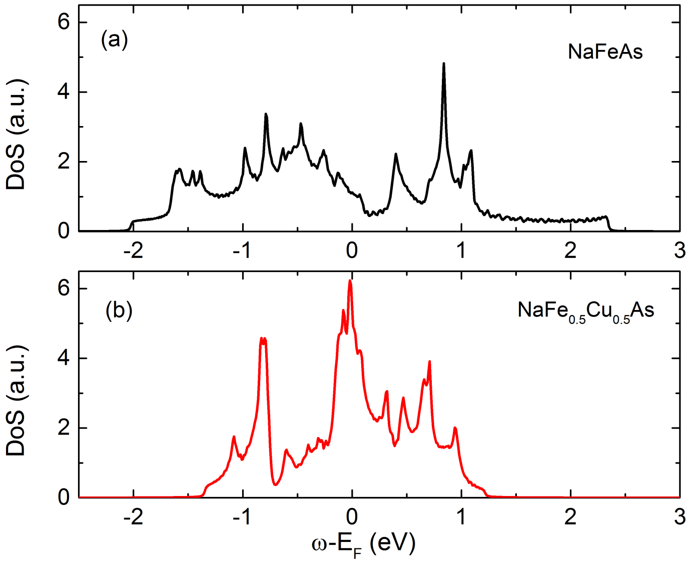

Supplementary Figure 11: Non-interacting density of states (DoS)ofthe models for (a) NaFeAs and (b) $NaFe_{0.5}Cu_{0.5}As$, respectively.

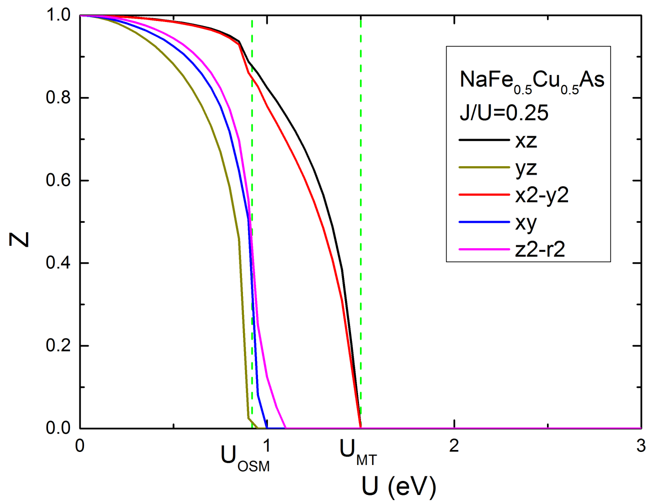

Supplementary Figure 12: Calculated orbital-resolved quasiparticle spectral weights of the multiorbital Hubbard model for $NaFe_{0.5}Cu_{0.5}As$. Here, we have taken the electron filling n=5 per Fe ion and J/U=0.25.

Atom	Site	X	y	z	Occupancy	U_{iso}(10^{-2}Å)
Na	8j	0.17498(52)	0.23423(70)	0	1.0	1.11(18)
As	8j	0.89665(27)	0.25595(36)	0	1.0	0.73(15)
Fe1	4b	0.5	0	0.25	1.03(13)	0.47(10)
Cu1	4b	0.5	0	0.25	-0.03(13)	0.47(10)
Fe2	4a	0	0	0.25	0.26(11)	1.00(13)
Cu2	4a	0	0	0.25	0.74(11)	1.00(13)

Supplementary Table 1: Structure of $NaFe_{0.56}Cu_{0.44}As$ with Fe-Cu stripe order with the space group *Ibam* from single crystal neutron diffraction refinement at 250K. The notation used is different from Fig. 1(d) in main text.The fit lattice parameters are a = 13.85(2) Å, b = c= 5.723(9) Å. Twin 1 scale factor 1.821(78), twin 2 scale factor 0.960(59). R_f = 3.13%, R_{f2} = 4.46%,χ^2 = 0.794.

Q_{twin1}	Q_{twin2}	I_{obs}	I_{calc}
1 -1 0	1 0 -1	0.61(6)	0.5970
-1 0 1	-1 1 0	0.35(7)	0.3175
3 0 1	3 1 0	0.31(10)	0.5661
5 1 2	5 2 1	1.82(21)	1.9646
9 2 3	9 3 2	3.56(31)	3.5543
9 3 4	9 4 3	0.70(20)	0.5188
1 1 0	1 0 1	0.66(6)	0.5970
7 2 1	7 1 2	0.38(13)	0.5404
3 3 2	3 2 3	2.85(26)	2.7429
5 4 3	5 3 4	4.92(35)	3.9764
9 4 3	9 3 4	0.74(27)	0.8646
7 5 4	7 4 5	1.07(35)	0.6652
5 3 0	5 0 3	5.07(29)	5.5651
15 3 0	15 0 3	0.92(25)	1.4295
9 4 1	9 1 4	1.27(35)	2.1441
13 4 1	13 1 4	2.03(58)	3.9126
1 5 2	1 2 5	1.39(28)	1.0085
5 5 2	5 2 5	12.75(67)	10.6521
9 5 2	9 2 5	1.34(34)	1.3046
1 6 3	1 3 6	5.95(72)	5.8215
5 6 3	5 3 6	0.96(38)	1.5489
7 5 0	7 0 5	0.62(30)	0.4075

Supplementary Table 2: Measured and calculated super-lattice peak intensities for$NaFe_{0.56}Cu_{0.44}As$ with the space group *Ibam* from single crystal neutron diffraction. Due to twinning measured intensity at (H, K, L) has contributions from (H, K, L) from twin 1 and (H, L, K) from twin 2. Peaks with no measurable intensities are not listed.

Sample/lattice parameter	Atom	Site	*x*	*y*	*z*	Occupancy	U_{iso} (10^{-2}Å)
$NaFe_{0.984}Cu_{0.016}As$ *a* = 3.9546(1) Å *c* = 7.0373(3) Å	Na	2c	0.25	0.25	0.3555(7)	1	1.9(1)
	Fe	2a	0.75	0.25	0	0.984	1.25(4)
	Cu	2a	0.75	0.25	0	0.016	1.25(4)
	As	2c	0.25	0.25	0.7973(4)	1	1.33(6)
$NaFe_{0.82}Cu_{0.18}As$ *a* = 3.9985(1) Å *c*=6.9932(3) Å	Na	2c	0.25	0.25	0.3554(4)	1	1.61(7)
	Fe	2a	0.75	0.25	0	0.82	1.14(4)
	Cu	2a	0.75	0.25	0	0.18	1.14(4)
	As	2c	0.25	0.25	0.7954(2)	1	1.09(5)
$NaFe_{0.61}Cu_{0.39}As$ *a* = 4.0353 (2) Å *c*=6.9468(5) Å	Na	2c	0.25	0.25	0.3601(7)	1	1.6(1)
	Fe	2a	0.75	0.25	0	0.61	1.12(7)
	Cu	2a	0.75	0.25	0	0.39	1.12(7)
	As	2c	0.25	0.25	0.7960(3)	1	1.04(7)
$NaFe_{0.56}Cu_{0.44}As$ *a* = 4.0472(1) Å *c* = 6.9262(3) Å	Na	2c	0.25	0.25	0.3601(7)	1	1.6(1)
	Fe	2a	0.75	0.25	0	0.61	1.12(7)
	Cu	2a	0.75	0.25	0	0.39	1.12(7)
	As	2c	0.25	0.25	0.7960(3)	1	1.04(7)

Supplementary Table 3: Refinement results from neutron powder diffraction data on $NaFe_{1-x}Cu_xAs$. All measurements are taken at 300 K and are fit using *P4/nmm* space group as super-lattice peaks are too weak to be seen in powder diffraction data. For $NaFe_{0.984}Cu_{0.016}As$, *Rp* = 5.08%, *wRp* = 6.38% and χ^2 = 1.130. For $NaFe_{0.82}Cu_{0.18}As$, *Rp*= 4.20%, *wRp* = 5.14% andχ^2 = 1.270. For $NaFe_{0.61}Cu_{0.39}As$, *Rp*= 4.11%, *wRp* = 5.02% andχ^2 = 1.123. For $NaFe_{0.56}Cu_{0.44}As$, *Rp* = 4.79%, *wRp* = 5.97%, χ^2 = 1.098.

Supplementary Note 1: Fe and Cu ordering in $NaFe_{1-x}Cu_xAs$ as seen in diffraction measurements

The crystal structure of $NaFe_{0.56}Cu_{0.44}As$ obtained from single crystal neutron diffraction refinement is shown in Supplementary Table S1. We collected 120 reflections at Bragg peaks associated with the NaFeAs structure and measured 95 super-lattice peak positions. 22 super-lattice peaks with measurable intensities are identified (Supplementary Table S2), these peaks are refined together with Bragg peaks associated with the NaFeAs structure (not shown). Due to twinning the measured intensity at (H,K,L) have contributions from (H,K,L) from twin 1 and (H,L,K) from twin 2. Population of the twins is roughly 2:1 for this particular sample. Due to formation of Fe-Cu stripes, y positions of Na and As also shift from their high symmetry position 0.25 by δ_{Na} and δ_{As}, respectively.

The crystal structure for $NaFe_{0.56}Cu_{0.44}As$ in Supplementary Table 1 approximates the ideal structure of $NaFe_{0.5}Cu_{0.5}As$ as shown in Figure 1(d), where Fe and Cu order into stripes forming a structural analog of the stripe magnetic order in NaFeAs. This reduces the symmetry of the system to *Ibam*space group. We adopt a unit cell similar to the orthorhombic structural unit cell of NaFeAs throughout the rest of paper as shown in Figure 1(d) unless otherwise stated. Compared to the *Ibam* notation used in Supplementary Table 1,a, b and c in this notation correspond to b, c, and half of a in the *Ibam*notation.Inthe notation used in Figure 1(d), super-lattice peaks due to Fe-Cu ordering occur at (H,K,L) with H = 1, 3, 5…, K = 0, 2, 4… and L = 0.5, 1.5, 2.5…. Magnetic peaks in this notation occur at (H,K,L) with H = 0, 2, 4…, K = 1, 3, 5… and L = 0.5, 1.5, 2.5… and (H,K,L) with H = 1, 3, 5…, K = 1, 3, 5… and L = 0, 1, 2….

To illustrate the effect of Fe-Cu ordering seen in neutron diffraction experiments, calculated neutron powder diffraction profiles for $NaFe_{0.5}Cu_{0.5}As$ with (i) disordered Fe and Cu and $\delta_{As}= \delta_{Na} = 0$, (ii) ordered Fe and Cu and $\delta_{As}= \delta_{Na} = 0$ and (iii) ordered Fe and Cu with $\delta_{As}= \delta_{Na}= -0.01$ are compared in Supplementary Figure 1 assuming $b = c$(*Ibam* notation). With this assumption, case (i) becomes tetragonal with *P4/nmm* symmetry. In case (ii) where Fe-Cu order into stripes, the only effect is to induce super-lattice peaks without affecting the intensity of nuclear Bragg peaks already present in case (i). For case (iii), introducing non-zero δ_{As}andδ_{Na}, nuclear Bragg peaks already present in case (i) change only slightly.

Given these considerations and for $NaFe_{1-x}Cu_xAs$ with x<0.5 the already weak super-lattice peaks become weaker and broader with decreasing doping [Supplementary Figure 5(f)]weuse *P4/nmm* space group appropriate for NaFeAs in the tetragonal state [1] to fit the our room temperature neutron powder diffraction (NPD) data for $NaFe_{1-x}Cu_xAs$. Doing so the information related to Fe-Cu ordering is neglected but other aspects of the structure can still be reliably obtained. Sincethe Fe-Cu ordering exists already at room temperature [Figure 2(c)], it will be interesting in future work to see if an order-disorder transition occurs at elevated temperatures where *P4/nmm* symmetry can be recovered with Fe and Cu becoming disordered and $\delta_{As}= \delta_{Na} = 0$ corresponding to case (i).

The NPD refinementresultsfor $NaFe_{1-x}Cu_xAs$ with x = 0.016, 0.18, 0.39 and 0.44 at room temperature are shown in Supplementary Figures2(a)-(d) and the refined structuralparameters are shown in Supplementary Table 3. Na and As occupancies are assumed to be 1, refinements of their occupancies also yield values close to 1. Given the similar scattering lengths of Fe and Cu, the occupancies of these two elements are set to values determined from ICP.The NPD data at 4K for $NaFe_{1-x}Cu_xAs$ with x = 0.44 is shown in Supplementary Figure 2(e). Only one super-lattice/magnetic peak is clearly seen as shown in the inset, so it is not possible to reliably refine δ_{As} and δ_{Na} from NPD data. Instead this data is used to estimate size of the ordered moment, refining the ordered moment for the magnetic structure in Figure 1(d) assuming Fe-Cu ordering we find 1.12(9)μ_B[Supplementary

Figure 2(e)], whereas assuming disordered Fe and Cu we obtain 1.4(1)μ_B.(0,0,1), (1,0,1) and (1,1,0) shown in the inset of Supplementary Figure 2(e) are in *Ibam*notation[Supplementary Tabel 1], corresponding to (0,1,0), (0,1,0.5) and (1,0,0.5) respectively in the notationof Figure 1(d).

Supplementary Note 2: XAS and RIXS measurements on the valence of Cu in $NaFe_{0.56}Cu_{0.44}As$

XAS for $NaFe_{1-x}Cu_xAs$ (x = 0.44) is shown in Supplementary Figure 3(a) for both π and σ polarizations [2], compared with XAS of $SrCuO_2$ (dashed line) measured with identical experimental configuration. The absorption peak at 930.7eV in $SrCuO_2$due to Cu^{2+} is absent in $NaFe_{1-x}Cu_xAs$(x = 0.44), demonstrating Cu^{1+} valence in heavily Cu-doped $NaFe_{1-x}Cu_xAs$ samples. Previous results on Cu in different valence states [3]showa chemical shift of ~2 eV between the absorption peaks for $Cu^{1+}$and Cu^{2+}.Thisagrees with our observation that the sharp absorption peak (931.8 eV) in $NaFe_{1-x}Cu_xAs$ (x = 0.44) is higher than the Cu^{2+} absorption peak in $SrCuO_2$, suggesting that Cu in $NaFe_{1-x}Cu_xAs$ assumes the Cu^{1+}oxidation state. The two overlapping peaks at ~ 935.5 eV and 936.5 eV are likely due to traces of unreacted elemental Cu. We have further searched for magnetic excitations with resonant inelastic X-ray scattering (RIXS)CuL_3 edge, but did not observe indication for magnetic modes for any of the sampled momentum transfers, typical scans at Q = (0.23,0.23) are shown in Supplementary Figure 3(b).The absence of magnetic excitations is expected for Cu^{1+} with a Cu $3d^{10}$ electronic configuration. Thus, both XAS and RIXS results confirm that Cu is in the nonmagnetic Cu^{1+} state and the elastic magnetic order observed by neutron scattering is entirely due to Fe.

Supplementary Note 3: Additional neutron scattering results

The ordered moment sizes were estimated from normalizing rocking scans of magnetic peaks against a weak nuclear Bragg peak (2, 0, 0) in single crystal elastic neutron scattering measurements. While super-lattice peaks occur at (1, 0, 0.5) and equivalent wave-vectors and magnetic peaks occur at (0, 1, 0.5) and equivalent positions, they overlap in reciprocal spacedue to twinning. Supplementary Figure 4 shows scans along the [H, 0, L]/[0, K, L] directions at 3.6 K and 300 K for the x = 0.44 sample. In Supplementary Figure5(a), the **Q**-dependence of magnetic peak intensities are shown, the magnetic intensities are obtained from rocking scans at 3.6 K after correcting for the super-lattice contributions using the ratio of peak intensities at 3.6 K and 300 K shown in Supplementary Figure4 and the Lorentz factor. In Supplementary Figure5(b)-(c), the calculated **Q** dependence of magnetic peak intensities are plotted for spins oriented along a, b and c assuming Fe^{2+} magnetic form factor. Comparing the measured **Q**-dependence in Supplementary Figure5(a) and the calculated **Q**-dependence in Supplementary Figure5(b)-(c), we conclude that the ordered magnetic moments are oriented predominantly along the *a*axis.

Supplementary Figure5(d) shows the wave vector dependence of the super-lattice peaks at 300 K, obtained from scans along the [H,0,L]/[0, K, L] directions in Supplementary Figure4.This experimental dependence can be qualitatively described by the structure for $NaFe_{0.56}Cu_{0.44}As$ in Supplementary Table 1, as shown Supplementary Figure5(e). For $NaFe_{1-x}Cu_xAs$ with$x$ = 0.39,both the magnetic peak [Figure3(a)]and the super-lattice peak [Supplementary Figure5(f)]are short-range and have similar widths, suggesting the magnetic signal seen arises from Fe-Cu stripe order.

To conclusively determine the orientation of the ordered magnetic moment, we carried out detailed polarized neutron scattering experiments. Since in our notation magnetic peaks occur at (0, K, L) with K = 1, 3, 5… and L = 0.5, 1.5, 2.5… but not (H, 0, L) positions, we only see magnetic signal from [0, K, L] scattering plane. We

define neutron polarization directions along momentum transfer **Q** as x, perpendicular to **Q** but in the [0, K, L] scattering plane as y and perpendicular to the scattering plane as z as shown in Supplementary Figure6(a). As our neutron scattering samples have twin domains, we cannot distinguish the (H, 0, L) from the (0, K, L) positions in these measurements.

Since SF neutron diffraction is only sensitive to spin components perpendicular to both momentum transfer $\boldsymbol{Q}$ and neutron polarization direction, one can conclusively determine the spin components along all crystallographic axes M_a, M_b and $M_c$from the observed magnetic peaks. M_a, M_b and M_c can be obtained via, $\sigma_x^{SF} - \sigma_y^{SF} \propto M_y = \sin^2\theta M_b + \cos^2\theta M_c$ and $\sigma_x^{SF} - \sigma_z^{SF} \propto M_z = M_a$, where σ_x^{SF}, σ_y^{SF} and σ_z^{SF} are neutron SF scattering cross sections with polarization directions along x, y and z, respectively, and θ is the angle between momentum transfer **Q** and (0, 1, 0). Supplementary Figure 6(b) shows the wave vectors probed in [0, K, L] scattering plane and the corresponding θ. In all cases $M_z = M_a$ while for momentum transfer with small θ, M_y is most sensitive to M_c and for momentum with larger θ, M_y is most sensitive to M_b. Measuring at the momentum transfers shown in Supplementary Figure 6(b), it can be seen in Supplementary Figure 6(c)-(f) that a clear peak can be seen for M_z but no peak is seen for M_y in all cases. These results conclusively show that spins are along the a axis, with negligible spin components along the other two directions, in agreement with our conclusion from unpolarized neutron diffraction results in Supplementary Figure 5(a)-(c).

In Supplementary Figure7(a)-(b), the temperature dependence of magnetic and super-lattice peaks of $NaFe_{0.56}Cu_{0.44}As$ are shown for **Q** = (1,0,0.5)/(0,1,0.5) and **Q** = (1,1,0) respectively.At both wave-vectors, the SF channel shows magnetic signal with T_N ~ 200K. At **Q** = (1,0,0.5), the super-lattice peak appears in the NSF channel and is weakly temperature dependent. At **Q** = (1,1,0), there is no signal in the NSF channel below 200K, consistent with this wave-vector being forbidden in the structures ofNaFeAs and $NaFe_{0.5}Cu_{0.5}As$ in Figure 1(c) and (d).

Supplementary Note 4: Electronic Structure Calculations for $NaFe_{0.5}Cu_{0.5}As$

Paramagnetic phase:

We first studied the non-magnetic phase of $NaFe_{0.5}Cu_{0.5}As$, using the space group *Ibam* deduced from the high-resolution TEM and single crystal neutron diffraction measurements [Supplementary Table 1]. The electronic density of states calculated with DFT-GGA method is shown in Supplementary Figure 9(b). The calculations indicate that $NaFe_{0.5}Cu_{0.5}As$ is expected to be a good metal, with the DOS at the Fermi level of 2.5 states per formula unit (f.u.). It is instructive to compare this value to the DOS in the parent compound NaFeAs [Supplementary Figure 9(a)], which is actually lower at ~2.0 states per f.u. This is understood because in NaFeAs, the Fermi level lies near a trough of the DOS, whereas in $NaFe_{0.5}Cu_{0.5}As$, it sits of the shoulder of the Fe d-state peak. This is also consistent with the fact that the Fe d-electron bandwidth is narrower than in NaFeAs, resulting in a larger DOS.

At first sight, the theoretical finding of the metallic behavior is puzzling, given the experimental observation of the insulating nature of $NaFe_{0.56}Cu_{0.44}As$. Conceivably, this may be an indication of the failure of the *ab initio* theory, since strong electron correlations are notoriously difficult to capture with DFT. To estimate the effect of the Coulomb repulsion U, we have performed a DFT+U calculation [4] on $NaFe_{0.5}Cu_{0.5}As$ using the interaction strength U = 3.15 eV and Hund's coupling J = 0.4 eV calculated in Ref. [5]. Curiously, although Coulomb repulsion does reduce the DOS by creating a pseudogap like feature at the Fermi level, it still predicts $NaFe_{0.5}Cu_{0.5}As$ to be a metal (DOS(E_F) ~2.1 states/f.u.) rather than an insulator in the paramagnetic state, as shown in Supplementary Figure 9(c). This is in stark contrast to the experimentally measured resistivity, which

exhibits insulating behavior even in the paramagnetic phase, above the AF ordering temperature in $NaFe_{0.5}Cu_{0.5}As$ [see Figure 2(f) in the main text]. Clearly, strong electron correlations are present that are not captured properly by the DFT or DFT+U calculations in the paramagnetic phase.

Antiferromagnetic phase:
We have also calculated the AF configuration of $NaFe_{0.5}Cu_{0.5}As$, which turns out to be more stable than the paramagnetic state by 0.28 eV/f.u. in our DFT-GGA calculations. We note that the antiferromagnetic order lowers the symmetry of the paramagnetic *Ibam* space group down to its subgroup *I222*. The Fe magnetic moments order in the (*ab*)-plane according to the experimentally observed **Q** = (1,0) wave-vector, as depicted schematically in the left panel of Supplementary Figure 8. The value of the ordered moment is predicted by DFT to be 2.77 μ_B on Fe site, larger than the experimentally measured ordered moment of ~1.4 μ_B per Fe. We find that the Cu moment is essentially zero (~0.02 μ_B), consistent with the full shell Cu^{1+} (d^{10}) configuration inferred from the experiments. Calculations within the DFT-GGA formalism show that the electronic density of states at the Fermi level is severely suppressed in the AF state, as shown in Supplementary Figure 10(a). Including the effect of the Coulomb repulsion using the DFT+U approach opens up a spectral gap of about 0.1 eV at the Fermi level, resulting in the true *insulating* state. The DFT+U also predicts Cu ions to be non-magnetic, while stabilizing an even higher ordered moment on Fe sites (3.29 μ_B per Fe).

It is instructive to compare the *ab initio* AF calculations with the experiment. In DFT and DFT+U calculations, the entire ordered moment comes from Fe site, while Cu ions are non-magnetic. While this is consistent with our experimental results, the predicted moment is much larger (2.77 μ_B in GGA) than the experimentally measured value of ~ 1.4 μ_B.

In conclusion, theoretical electronic structure calculations based on DFT and DFT+U indicate that the value of Coulomb repulsion is not large enough by itself to result in the paramagnetic Mott insulating state in $NaFe_{0.5}Cu_{0.5}As$, which appears to contradict the observed insulating behavior of resistivity [Fig. 1(f) in the main text]. Allowing the possibility of an AF ordering amplifies the effect of Coulomb interaction, resulting in an insulating magnetic ground state. However, the theory predicts the average value of the ordered moment per Fe site too large compared with the experimental value from neutron diffraction. This deficiency of DFT in exaggerating the ordered Fe moment is well documented in other materials in the iron pnictide family [5, 6]. The electronic structure calculations do capture correctly an enhanced propensity to AF ordering when compared to the parent compound NaFeAs, which has a very small ordered moment ~0.1 μ_B/Fe [1]. This is consistent with the electronic correlations becoming stronger upon Cu doping.

Supplementary Note 5: Effects of electron correlations

An important consequence of the large local potential difference between Cu and Fe is the kinetic blocking mechanism: the hopping process between Cu and Fe ions are substantially reduced in $NaFe_{0.5}Cu_{0.5}As$ compared to that between Fe-Fe ions in NaFeAs.Within our approximation, such a kinetic blocking mechanism completely suppresses the inter Fe-Cu and intra Cu-Cu hoppings. This effectively reduces the kinetic energy. As for the hopping integrals between Fe-Fe, we adopt the same tight-binding parameters as in NaFeAs. With this setup, we have calculated the density of states in the non-interacting limit of models for NaFeAs and $NaFe_{0.5}Cu_{0.5}As$, respectively. As shown in Supplementary Figure 11, the overall bandwidth of the $NaFe_{0.5}Cu_{0.5}As$ is reduced from the one of NaFeAs by a factor about 41%. As we will discuss later in this section, this kinetic energy reduction is crucial to stabilizing a Mott insulating state in $NaFe_{0.5}Cu_{0.5}As$.

For NaFeAs, a metal-to-Mott-insulator transition takes place when U exceeds about 5 eV. For sufficiently large J/U, this transition involves a third intermediate orbital-selective Mott phase (OSMP), where the Fe $3d_{xy}$ orbital is Mott localized with zero quasiparticle spectral weight, while all other $3d$ orbitals are still itinerant. Note that similar OSMP has been found in a model for $K_xFe_{2-y}Se_2$ system [7]. In the metallic phase, there is a crossover from a weakly coupled metallic state to a strongly coupled one, at which the quasiparticle spectral weights drop rapidly. In the strongly coupled metallic state, the quasiparticle spectral weight shows strong orbital selectivity. Such a crossover is a quite general feature of the models for both iron pnictides and iron chalcogenides [7,8].

To estimate the strength of electron correlations in the NaFeAs system, we compare the bandwidth renormalization factor calculated from our theoretical model to the one obtained in ARPES experiments. In Ref. [9], an overall bandwidth renormalization factor of 4 has been reported. Here we estimate the J and U values of the system by requiring the theoretically calculated renormalization factor $1/Z_\alpha$ for each orbital α between 2 to 6. We then assume that the J and U values in the NaFeAs and $NaFe_{0.5}Cu_{0.5}As$ systems are comparable. These estimated parameter values form a "physical" parameter regime in the phase diagram, which is shown as the shaded regime in Fig.4(c) of the main text.

By treating the Cu ions as vacancies, we obtain the phase diagram of the model for $NaFe_{0.5}Cu_{0.5}As$, as shown in Fig.4(c) of the main text. The evolution of quasiparticle spectral weights with U at J/U=0.25 for $NaFe_{0.5}Cu_{0.5}As$ is shown in Supplementary Figure 12. There are three phases, a metallic one, an OSMP, and a Mott insulator. Compared to the case of NaFeAs, the critical U for the Mott transition (U_{MT}) at any given J/U value is substantially reduced. This reduction of U_{MT} can be readily understood. As mentioned above, the local potential difference between the Cu and Fe sites leads to kinetic blocking, which reduces the kinetic energy. This effect is augmented by the valence of Fe being Fe^{3+} (with n = 5), which also originates from the local potential difference between the Cu and Fe sites. The reduction of U_{MT} in the model for $NaFe_{0.5}Cu_{0.5}As$ already pushes the "physical" parameter regime to lie inside the Mott insulating part of the phase diagram. Our theoretical result is consistent with our experimental one, which indicates that $NaFe_{0.5}Cu_{0.5}As$ is a Mott insulator.

Supplementary References

[1] Li, S. *et al.* Structural and magnetic phase transitions in $Na_{1-\delta}FeAs$, *Phys. Rev. B***80**, 020504(R) (2009).
[2] Zhou, K.-J.*et al.* Persistent high-energy spin excitations in iron-pnictide superconductors, *Nat. Commun.***4**, 1470 (2013).
[3] Jiang, P. *et al.* Experimental and theoretical investigation of the electronic structure of Cu_2O and CuO thin films on Cu(110) using x-ray photoelectron and absorption spectroscopy, *J. Chem. Phys.***138**, 024804 (2013)
[4] Anisimov, V. I., Zaanen, J. & Andersen, O. K. Band theory and Mott insulators: Hubbard*U*insteadofStoner*I*, *Phys. Rev. B***44**, 943-954 (1991).
[5] T. Miyake, K. Nakamura, R. Arita, & M. Imada, J. Phys. Soc. Jpn. **79**, 044705 (2010).
[6] Mazin, I. I., Johannes, M. D., Boeri, L., Koepernik,K. & Singh, D. J. Problems with reconciling density functional theory calculations with experiment in ferropnictides, *Phys. Rev. B***78**, 085104 (2008).
[7] Yu, R. & Si,Q. Orbital-Selective Mott Phase in Multiorbital Models for Alkaline Iron Selenides $K_{1-x}Fe_{2-y}Se_2$, *Phys. Rev. Lett.***110**, 146402 (2013).
[8] Yu, R. & Si, Q. *U*(1) slave-spin theory and its application to Mott transition in a multiorbital model for iron pnictides, *Phys. Rev. B***86**, 085104 (2012).
[9] Yi, M. *et al.* Electronic reconstruction through the structural and magnetic transitions in detwinned NaFeAs, *New J. Phys.***14**, 073019 (2012).
